# Discovery of regulatory complexes in Arabidopsis via protein–metabolite mapping during *Pseudomonas* infection

**DOI:** 10.1093/plphys/kiag349

**Published:** 2026-06-09

**Authors:** Jieun Kang, Ewelina M Sokolowska, Hanne Zillmer, Talia Karasov, Dirk Walther, Aleksandra Skirycz

**Affiliations:** Department of Biochemistry and Molecular Biology, Michigan State University, East Lansing, MI 48824, United States; Institute of Molecular Plant Physiology, Max-Planck-Institute of Molecular Plant Physiology, Potsdam-Golm 14476, Germany; Institute of Molecular Plant Physiology, Max-Planck-Institute of Molecular Plant Physiology, Potsdam-Golm 14476, Germany; School of Biological Sciences, University of Utah, Salt Lake City, UT 84112, United States; Institute of Molecular Plant Physiology, Max-Planck-Institute of Molecular Plant Physiology, Potsdam-Golm 14476, Germany; Department of Biochemistry and Molecular Biology, Michigan State University, East Lansing, MI 48824, United States

## Abstract

Protein–metabolite interactions (PMIs) are critical regulators of cellular processes, yet their roles in plant–pathogen interactions remain poorly understood. Here, we applied PROMIS (Protein–Metabolite Interactions using Size separation) to map the Arabidopsis protein–metabolite interactome during infection with *Pseudomonas syringae* pv. *tomato* DC3000 (*Pto* DC3000). PROMIS resolved elution profiles for >5,700 proteins and 192 annotated metabolites across infected, systemic, and control tissues, revealing infection-associated remodeling of protein assemblies and metabolite co-fractionation patterns. Integrating co-fractionation with coronatine-induced transcriptional responses, we identified NATA1, a polyamine *N*-acetyltransferase, as a coronatine-associated host protein binder. Biochemical assays confirmed direct coronatine–NATA1 interaction (Kd = 586 nm), whereas the homolog NATA2 did not bind coronatine. Coronatine did not strongly inhibit NATA1 catalytic turnover under our assay conditions but consistently depressed NATA1 dimer formation in vitro and in planta. In silico docking together with targeted mutational analyses identified a coronatine-sensitive interface on NATA1, revealing residue-specific coupling between ligand binding, assembly state, and acetyl-CoA engagement. Collectively, these findings establish PROMIS as a robust platform for infection-state PMI discovery and uncover a coronatine-responsive regulatory node that links small-molecule signaling to host polyamine acetylation.

## Teaser

Mapping molecular interactions in infected plant revealed how a bacterial toxin reshapes host regulation during disease.

## Introduction

Protein–metabolite interactions (PMIs) represent a fundamental layer of cellular regulation, integrating metabolic and signaling networks to shape biological outcomes. While proteins, including enzymes, receptors, and transcription factors, are central to executing cellular functions, small molecules act as dynamic modulators, influencing protein activity, localization, and stability. Increasingly, metabolites are recognized not only as substrates or cofactors but as signaling entities that engage in direct physical interactions with proteins, forming regulatory complexes that fine-tune cellular responses (Kosmacz et al. 2020; Venegas-Molina et al. 2021; Wagner et al. 2021; Gasperini and Howe 2024). Despite advances in identifying small molecule signals, the mechanisms by which PMIs operate, particularly at the interface of interorganismal interactions, remain poorly understood.

Plant–pathogen interactions offer a robust framework for studying molecular crosstalk (Xin and He, 2013). In these encounters, a host and a microbe engage in a biochemical tug-of-war, deploying a sophisticated arsenal of proteins and metabolites to outmaneuver each other. In *Arabidopsis thaliana*, the perception of pathogen-associated molecular patterns (PAMPs) by pattern recognition receptors (PRRs), such as flagellin sensing 2 (FLS2), triggers basal immune response known as PAMP-triggered immunity (PTI) (Yuan et al. 2021). While much of the downstream signaling cascade is mediated by protein–protein interactions (PPIs) (Wang et al. 2022), mounting evidence highlights the importance of small molecules in determining the outcome of host–pathogen interactions. Plant responses to microbial pathogens are orchestrated by complex hormonal networks, including ethylene, salicylic acid (SA), and jasmonic acid (JA), and as well as specialized immune-related metabolites, such as *N*-hydroxy-pipecolic acid (Pang et al. 2021; Cai and Aharoni 2022; Monte 2023). In addition to these signaling molecules, primary metabolites, including sugars, amino acids, and lipids, are dynamically reprogrammed during infection, contributing to metabolic shifts and the activation of defense responses (Rojas et al. 2014). Specialized metabolites, such as glucosinolates and the phytoalexin camalexin, function as antimicrobial agents that impair bacterial colonization (Piasecka et al. 2015). Together, these layers of metabolic and hormonal regulation shape the plant's immune landscape and its ability to adapt to biotic stress. Equally, microbes can perceive and respond to host-derived specialized metabolites, including flavonoids, terpenoids, steroids, and phenolic compounds, which can either promote or restrict microbial colonization (Pang et al. 2021; Ketehouli et al. 2025). Moreover, microbial pathogens deploy their own small molecule arsenal to facilitate infection and suppress host immunity. For instance, the microbial virulence factor phevamine A impairs host polyamine synthesis, interfering with a pathway that generates key defense-associated metabolites, such as putrescine, spermidine, and spermine, known to support redox homeostasis and membrane integrity (O’Neill et al. 2018). Another phytotoxin, coronatine, mimics the active form of JA-Ile and directly targets the JA receptor CORONATINE INSENSITIVE 1 (COI1), forming one of the most well-characterized examples of a protein–metabolite complex in plant–microbe interactions (Katsir et al. 2008). Both phevamine A and coronatine are secreted by *Pseudomonas syringae* pv. *tomato* DC3000 (*Pto* DC3000), a model bacterial pathogen extensively used to investigate the molecular mechanisms of microbial virulence and plant immune responses (Xin and He, 2013). The Arabidopsis–*Pto* DC3000 pathosystem thus provides an ideal and tractable model for exploring how small molecules shape host–pathogen dynamics. We hypothesized that *Pto* DC3000 infection induces extensive remodeling of the Arabidopsis protein–metabolite interactome, leading to the formation of regulatory complexes. To test this, we employed PROMIS (Protein–Metabolite Interactions using Size separation), a co-fractionation mass spectrometry (CF-MS) platform developed in our lab to identify native PMIs (Veyel et al. 2017, 2018).

CF-MS methods combine biochemical separation of native complexes with mass spectrometry–based analysis of the resulting fractions and infer interactions from the similarity of elution profiles across chromatographic separations (Schlossarek et al. 2023). Such approaches have been instrumental in generating comprehensive PPI networks in both model and nonmodel organisms (Goel et al. 2024). In plants, CF-MS strategies have been applied across a wide range of tissues and subcellular compartments. Publicly available datasets include protein interactomes from mature Arabidopsis leaves (Aryal et al. 2014) and developing rice aleurone tissue (Lee et al. 2021), membrane-associated protein complexes from Arabidopsis (Gilbert and Schulze 2019), and chloroplast stromal protein complexes, also from Arabidopsis (Olinares et al. 2010).

Beyond individual systems, a pan-species CF-MS analysis spanning 13 plant model and crop species revealed both conserved and lineage-specific features of the plant protein interactome, including the distinctive architecture of the plant tRNA multisynthetase complex (McWhite et al. 2020). In a complementary study, Lee and Szymanski (2021) demonstrated that variation in protein multimerization has the potential to drive neofunctionalization across diverse protein classes. More recently, CF-MS analysis uncovered rapid ethylene-dependent changes in protein abundance and complex composition, revealing dynamic protein assembly and disassembly events with functional relevance to hypocotyl development and early seedling growth (Lee et al. 2025). Collectively, these studies establish CF-MS as a powerful framework for detecting state-dependent remodeling of native assemblies, an assumption that also underpins PMI discovery by PROMIS.

The demonstrated success of CF-MS in resolving protein complex composition and state-dependent remodeling motivated our application of PROMIS to investigate PMIs during pathogen infection. PROMIS employs size-exclusion chromatography (SEC) (Veyel et al. 2018), in which metabolites bound to protein complexes co-elute in early, high-molecular-weight fractions, whereas unbound small molecules elute in later fractions. The resulting fractions are subjected to parallel proteomic and metabolomic analyses, and co-eluting proteins and metabolites are identified using similarity metrics, such as Pearson correlation (Sokolowska et al. 2019). To enable users with little or no data analysis background to rapidly identify co-fractionating molecules based solely on their fractionation profiles, we developed a web-based, intuitive tool termed PROMIsed (Schlossarek et al. 2021).

Recent PROMIS studies in Arabidopsis (Veyel et al. 2017, 2018), budding yeast (Luzarowski et al. 2021; Schlossarek et al. 2022), and the thermophilic fungus *Chaetomium thermophilum* (Li et al. 2021) reported hundreds of metabolic features co-separating with protein complexes. Furthermore, a comparative analysis of yeast across 3 distinct growth stages demonstrated that PROMIS can capture state-specific remodeling of the protein–metabolite interactome (Schlossarek et al. 2022). A primary advantage of CF-MS-based approaches for studying PMIs is their untargeted and exploratory nature, enabling the simultaneous detection of hundreds of metabolic features associated with protein complexes in a single experiment. In this way, PROMIS provides a global view of a PMI landscape, facilitating hypothesis generation and the prioritization of candidate metabolites and proteins for downstream functional analyses. In this study, we apply PROMIS to Arabidopsis–*Pto* DC3000 infection to capture infection-associated reorganization of protein–metabolite complexes, including potential interorganismal associations between host proteins and pathogen-derived metabolites.

A key limitation of PROMIS, however, is that individual metabolites often co-fractionate with large numbers of proteins, many of which likely reflect coincidental co-elution rather than bona fide interactions. Distinguishing true binders from background therefore remains a central challenge. Experimentally, this can be addressed by incorporating orthogonal separation strategies, such as ion-exchange chromatography, which rely on distinct physicochemical principles (Wagner et al. 2025). Complementary targeted methods, including thermal proteome profiling (Kosmacz et al. 2018; Moreno et al. 2021; Chodasiewicz et al. 2022), can further refine candidate interactions for selected metabolites. In addition, emerging in silico docking approaches, enabled by advances in AI-based protein structure prediction, offer promising avenues for interaction prioritization (Walther 2023). Finally, biologically informed filtering remains essential; for example, we previously prioritized enzymes within the metabolic pathways of co-eluting metabolites to identify potential feedback and feed-forward regulatory interactions (Veyel et al. 2018).

Here, specifically for coronatine, we integrated co-fractionation data with coronatine-induced transcriptional responses, reasoning that proteins induced during infection are more likely to participate in pathogen-responsive PMIs. In doing so, we identify a regulatory interaction between coronatine and NATA1 (*N*-acetyltransferase 1) (Adio et al. 2011; Lou et al. 2016), a cytosolic enzyme that plays a key role in polyamine metabolism by catalyzing the acetylation of putrescine, thereby regulating polyamine homeostasis.

## Results

### PROMIS is successful in resolving known protein–protein and protein–metabolite complexes

**Figure 1:**
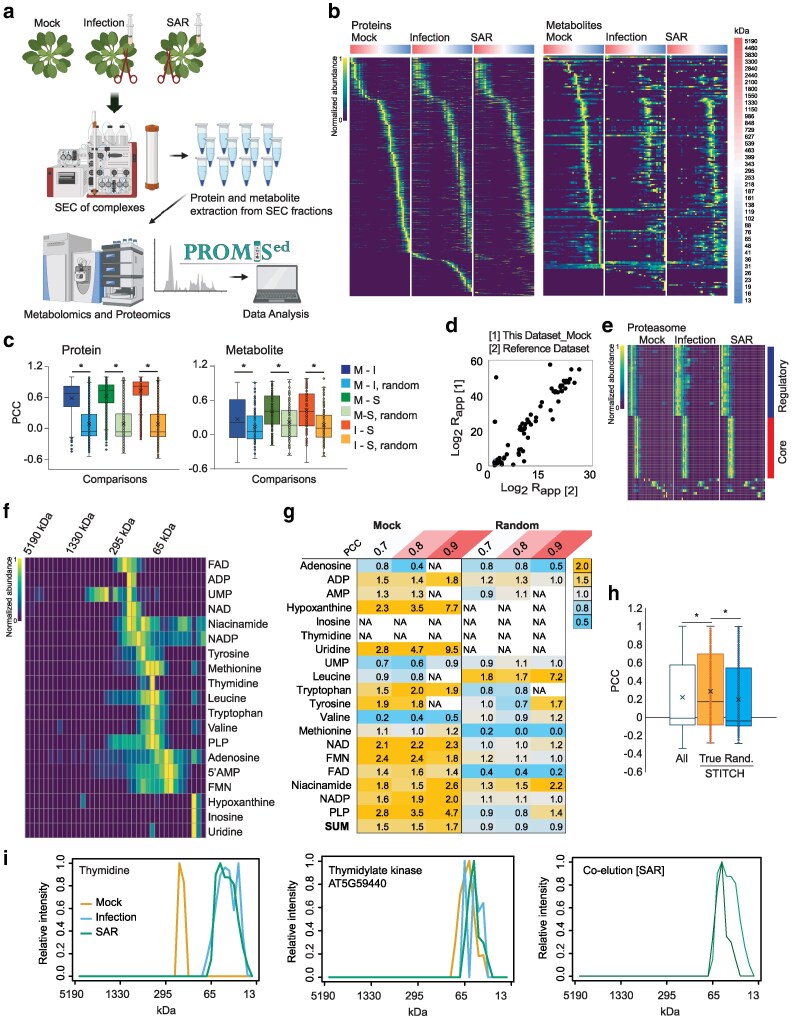
PROMIS separation of protein–protein and protein–metabolite complexes. a) Experimental design and PROMIS workflow for mock, locally infected, and distal SAR Arabidopsis leaves. Created using Biorender. b) Heat map representation of protein and metabolite elution profiles across the 39 SEC fractions for each condition. c) Similarity between profiles from mock and infected samples was assessed using PCC. To evaluate significance, PCC values were also calculated using randomized elution profiles. Statistical significance between true and randomized datasets was assessed using Student's *t*-test assuming equal variance and normal distribution. Asterisks indicate significance (*P* < 0.001). M, mock; I, infection; S, SAR. d) The distribution of R_app_ of proteins classified as PPI benchmarks in our dataset and in the reference dataset (Hayashi et al. 2023; Yang et al. 2025). e) Coordinated co-elution of subunits from 26S proteasome across mock, infection, and SAR separations. f to h) Known PMIs deposited in the STITCH database for 19 metabolites detected in the mock PROMIS dataset were used to calculate the sensitivity/1-specificity ratio for lists of putative protein interactors obtained from PROMIS using different PCC cutoffs. As an additional control, the same analysis was performed on a dataset in which protein elution profiles were randomized. f) Heat map visualization of the elution profiles of the metabolites used for the analysis. g) Sensitivity/1-specificity ratios for individual metabolites and calculated jointly (sum). h) Distribution of PCC calculated for all the protein–metabolite pairs in our dataset and for the subset of known interactions retrieved from STITCH and present in the mock separation for the true and randomized dataset. Statistical significance between true and randomized datasets was assessed using Student's *t*-test assuming equal variance and normal distribution. Asterisks indicate significance (*P* < 0.001). i) Example of condition-dependent recovery of an annotated interactor: thymidine co-fractionates with thymidylate kinase in infection and SAR datasets. c and h) Data are represented as box plot; the central line within each box represents the median. The lower and upper boundaries of the box indicate the first quartile (Q1) and third quartile (Q3), respectively, encompassing the interquartile range (IQR). Whiskers extend to the most extreme data points within 1.5 × IQR from the quartiles. Data points beyond this range are shown as outliers. Each box summarizes the variability and central tendency of the data for a given group. Analysis and visualization were performed using Microsoft Excel.

To investigate how *Pto* DC3000 infection reshapes native macromolecular assemblies, we performed PROMIS on mock-treated leaves, locally infected leaves, and distal leaves exhibiting systemic acquired resistance (SAR) (Fig. 1a). PROMIS fractionation yielded 39 fractions per condition spanning approximately 5 MDa to 10 kDa based on column calibration. Because this SEC column provides optimal resolution between ∼1.25 MDa and 10 kDa, higher-molecular-weight fractions eluting at or near the void volume were retained but interpreted cautiously with respect to size assignment.

Parallel proteomics and metabolomics quantified elution profiles for 5,744 proteins and 192 annotated metabolites (Fig. 1b; Datasets S1 and S2), including 909 *Pto* DC3000 proteins and 4,835 Arabidopsis proteins. To assess overall similarity across conditions, we compared elution-profile Pearson correlation coefficient (PCC) distributions for proteins and metabolites and contrasted these with randomized controls (Fig. 1c). The infection and SAR datasets exhibited greater similarity to each other than to the uninfected controls at both the protein and metabolite levels. Protein elution profiles were broadly similar across conditions, whereas metabolite profiles showed greater divergence, particularly between mock and infection. Together, these PCC distributions provide a global overview of condition-dependent changes in protein and metabolite elution behavior.

Because co-fractionation analyses assume that native assemblies are maintained throughout fractionation, we next assessed preservation of protein complexes during separation using an Arabidopsis benchmark set of PPIs (Yang et al. 2025). Using the mock dataset, we calculated the apparent multimerization ratio (Rapp) by comparing SEC-inferred apparent mass to monomeric mass (capped at 1.25 MDa). The majority of benchmark proteins (88/95) exhibited Rapp > 1.6, consistent with multimeric behavior under our conditions. Moreover, Rapp values derived from our mock dataset correlated strongly with those from the reference CF-MS–SEC dataset (Yang et al. 2025) (Pearson *r* = 0.89) (Fig. 1d). Consistent with this, multiple canonical assemblies displayed coordinated co-elution of subunits across mock and infection conditions, including the 19S regulatory particle and the 20S core particle of the 26S proteasome, COP9 signalosome, and the HCF-1 (host cell factor 1)/serine–threonine phosphatase complexes (Fig. 1e; Dataset S3). Together, these analyses support that native protein complex organization is largely retained through PROMIS fractionation.

Analogously to PPI analysis, we used the PCC between protein and metabolite elution profiles to evaluate PMI inference performance in our dataset. To select the optimal PCC cutoff, we examined co-elution patterns of 1,524 known PMIs retrieved from the STITCH database (experimental evidence, confidence score > 0.4) (Dataset S4). This analysis was performed using the mock dataset and encompassed 19 metabolites, including nucleosides/nucleotides, cofactors, and amino acids, representing a broad diversity of elution behaviors (Fig. 1f). For each metabolite individually, and for all interactions collectively, we calculated the positive likelihood ratio (sensitivity/(1 − specificity)) at 3 PCC thresholds: 0.7, 0.8, and 0.9. As a control, we generated a randomized dataset by shuffling protein identifiers (Fig. 1g). Overall, PCC = 0.9 yielded the highest positive likelihood ratio and thus provided the strongest enrichment for known interactions. However, it is important to note that all tested cutoffs enriched known interactors in the true, but not in the randomized, dataset. Correspondingly, comparison of PCC distributions for all proteins in the dataset versus the subset of known interactions revealed a significant shift toward higher PCC values in the true dataset relative to both the randomized control and the full protein set (Fig. 1h). As an example of metabolite-specific performance, we observed strong recovery for several cofactor interactomes, including PLP and NADH (Fig. 1g), whereas nucleosides/nucleotides and amino acids showed more variable recovery, which may reflect inherently weaker or more transient interactions. In addition, recovery of annotated interactors can be condition dependent. Notably, thymidine did not retrieve any of its 15 STITCH-deposited interactions under the mock condition. However, when examining infection and SAR datasets, thymidine co-fractionated with thymidylate kinase, one of its annotated interactors, under both conditions (Fig. 1i), illustrating that co-fractionation with known partners can be condition dependent.

Together, these analyses support the integrity of PROMIS fractionation and highlight that recovery of known interactions can be condition and metabolite dependent, necessitating further prioritization for targeted validation.

### Changes to protein complexes associated with *Pto* DC3000 infection

**Figure 2:**
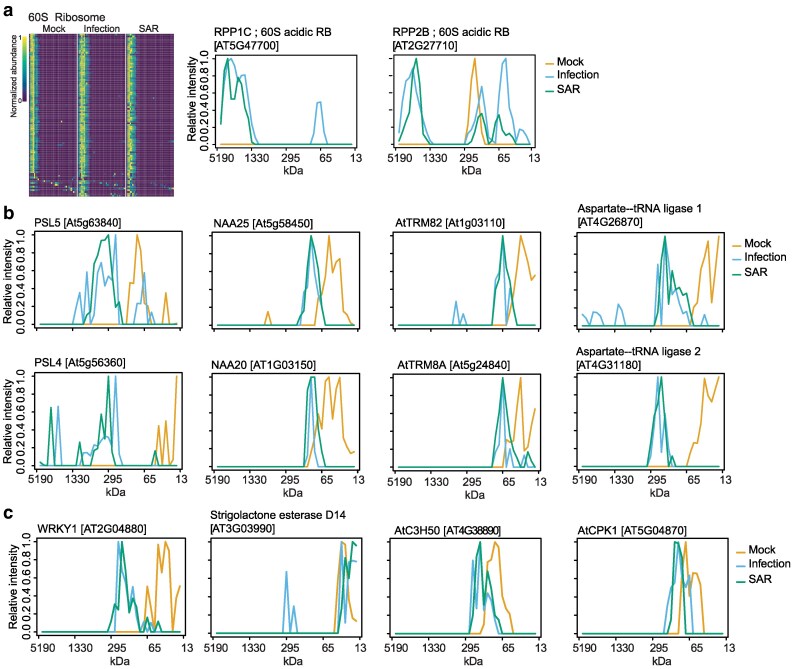
Infection-associated shifts in protein complex elution behavior. a) Representative elution profiles of 60S acidic ribosomal proteins across mock, infection, and SAR separations. AT5G47700 was primarily detected in infection/SAR samples, whereas AT2G27710 was present across conditions but displayed redistribution and additional peaks under infection/SAR. b) Elution profiles of previously reported PPIs recovered in PROMIS, specifically the PSL4–PSL5 complex, NatB complex, tRNA guanine-*N*(7)-methyltransferase complex, and the aspartyl-tRNA ligase 1/2 pair, where the change in the elution profile suggests differential assembly under infection/SAR conditions. c) Examples of regulatory proteins showing infection-dependent changes in elution behavior indicative of altered assembly state (WRKY1, strigolactone receptor D14, tRNA-dihydrouridine synthase AtC3H50, and calcium-dependent protein kinase 1 AtCPK1).

Although our primary goal was PMI mapping, the dataset also enables inspection of condition-dependent shifts in protein elution behavior. For example, several acidic ribosomal subunits (AT2G27710, AT4G00810, AT2G27720, AT1G01100, and AT5G47700) co-fractionated with the ribosome 60S complex specifically in the infection and SAR datasets (Fig. 2a). While some of the abovementioned 60S acidic ribosomal proteins were detected solely in infection/SAR samples, others were present across conditions but showed a redistribution and additional co-eluting peaks under infection/SAR, consistent with remodeling of ribosomal stalk-associated assemblies (Fig. 2a). This observation motivated us to examine whether similar condition-dependent co-fractionation shifts could be detected for additional, previously reported PPIs. Here, we highlight 4 previously reported PPIs that are detected across all 3 chromatographic separations, show infection- or SAR-dependent changes in their apparent multimerization state, and whose partners co-fractionate in both the infection and SAR conditions. These include the PSL4–PSL5 complex, the NatB complex, the tRNA guanine-*N*(7)-methyltransferase complex, and the aspartyl-tRNA ligase 1/2 complex (Fig. 2b). Notably, for each pair, the position of the dominant co-elution peak shifts toward fractions corresponding to higher-order assemblies in the infection and SAR profiles.

Four additional examples, WRKY1, the strigolactone receptor D14, tRNA-dihydrouridine synthase (AtC3H50), and calcium-dependent protein kinase 1 (AtCPK1), also exhibited infection-dependent shifts in elution behavior consistent with changes in assembly state. Although specific interacting partners could not be assigned from these data alone, these profiles further illustrate the ability of PROMIS to detect infection-responsive alterations in protein complex distribution (Fig. 2c).

Together, these observations demonstrate that PROMIS is capable of capturing condition-dependent changes in protein multimerization or complex distribution.

### Pathogen infection- and plant defense-associated metabolites engage with protein complexes

**Figure 3:**
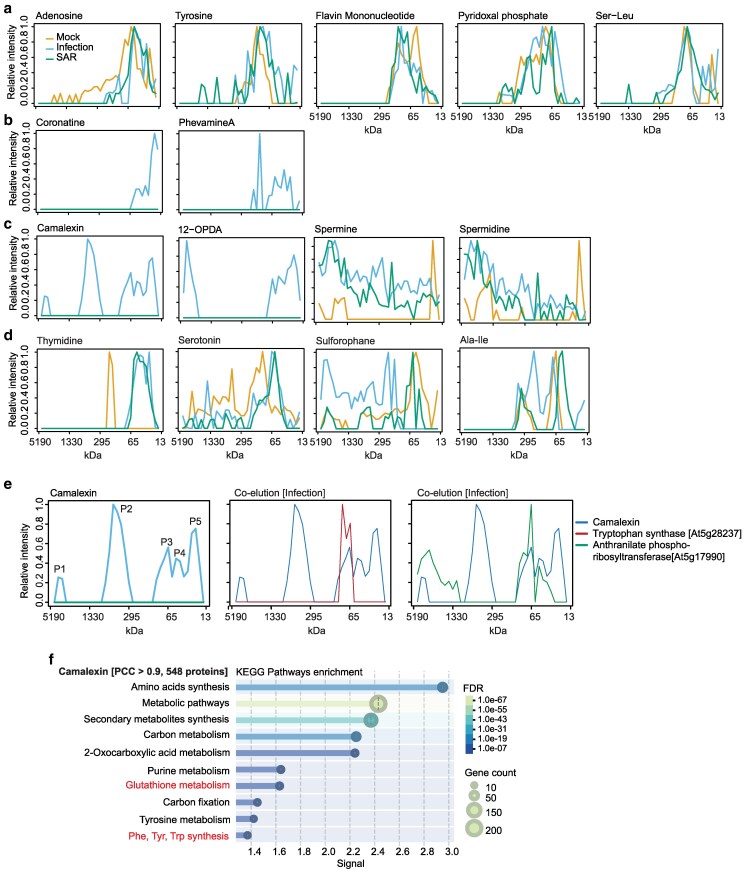
Infection-associated metabolite co-fractionation and candidate prioritization. a) Representative elution profiles of annotated metabolites spanning amino acids, nucleotides/nucleosides, cofactors, dipeptides, and specialized metabolites across mock, infection, and SAR separations. b) Elution profiles of *Pto* DC3000-derived virulence factors coronatine and phevamine A, detected exclusively in infected samples. c) Elution profiles of host defense-associated metabolites detected specifically in infected tissues (camalexin and 12-OPDA) and polyamines (spermine and spermidine) enriched in high-molecular-weight fractions under infection and SAR conditions. d) Examples of metabolites exhibiting condition-dependent shifts in elution behavior, including thymidine and serotonin (mock vs infection/SAR) and sulforaphane and the dipeptide Ala–Ile (infection vs both mock and SAR). e) Camalexin elution profile in infected samples showing multiple elution maxima and the corresponding candidate set obtained by PCC-based co-fractionation (PCC > 0.9). f) Functional enrichment analysis of the camalexin-associated candidate set highlighting enrichment for enzymes in tryptophan metabolism (eg tryptophan synthase and anthranilate phosphoribosyltransferase), illustrating pathway-informed prioritization.

In addition to resolving protein complexes, the PROMIS datasets included 192 annotated small molecules spanning amino acids, nucleotides/nucleosides, cofactors, dipeptides, and specialized metabolites. Of these, 153 metabolites were consistently detected across all 3 conditions (mock, infected, and SAR), and the majority of cofactors, amino acids, nucleotides/nucleosides, and dipeptides exhibited broadly comparable elution profiles across conditions (Fig. 3a).

We next focused on metabolites with condition-specific detection or clear changes in elution behavior in protein-containing fractions. Two *Pto* DC3000-derived virulence factors, coronatine and phevamine A, were detected exclusively in infected samples and eluted in protein-containing fractions (Fig. 3b). In addition, several host defense-associated metabolites also showed condition-dependent pattern. Camalexin and 12-oxo-phytodienoic acid (12-OPDA) were only detected in the infected tissues (Fig. 3c). Polyamines (spermine and spermidine) were enriched in high-molecular-weight fractions under infection and SAR conditions (Fig. 3c). Thymidine and serotonin also displayed differential elution behavior between mock and infection/SAR datasets (Fig. 3d). By contrast, sulforaphane and the dipeptide Ala–Ile showed elution patterns in infected samples that differed from both mock and SAR conditions (Fig. 3d).

To move from these condition-dependent elution patterns to specific candidate PMIs, we relied on PCC-based co-fractionation. However, as indicated in the Introduction, even when applying a relatively stringent PCC cutoff, we are still left with tens to hundreds of putative interactors per metabolite, making the identification of true binders a considerable challenge (Datasets S5 to S7). This highlights the need for explicit prioritization strategies that complement co-fractionation scoring, for example, by leveraging prior biological knowledge, integrating pathway context, or incorporating orthogonal datasets.

Camalexin provides an illustrative example of this challenge and a practical route toward prioritization. Camalexin exhibited a complex elution profile in the infection dataset with 5 distinct maxima (Fig. 3e), and co-fractionation analysis at PCC > 0.9 yielded 548 Arabidopsis proteins. Because camalexin is derived from tryptophan, we asked whether pathway context could help narrow candidates. Indeed, functional enrichment analysis of the camalexin-associated candidate set revealed enrichment for enzymes in tryptophan metabolism, including tryptophan synthase and anthranilate phosphoribosyltransferase (Fig. 3e and f), consistent with the idea that incorporating biosynthetic context can enrich for biologically plausible associations. We therefore used this general principle, co-fractionation followed by transparent, orthogonal prioritization, to select candidates for targeted validation in subsequent sections.

Together, these findings demonstrate that *Pto* DC3000 infection induces extensive remodeling of the Arabidopsis protein–metabolite interactome. The detection of both microbial virulence factors and host defense metabolites within protein-containing fractions underscores PROMIS's ability to capture infection-associated protein–metabolite associations. At the same time, the camalexin case illustrates that moving from global co-fractionation outputs to specific binders typically requires additional filtering and prioritization, motivating the candidate-selection framework applied below to coronatine-associated proteins.

### Discovery of NATA1 as a coronatine-binding protein

**Figure 4:**
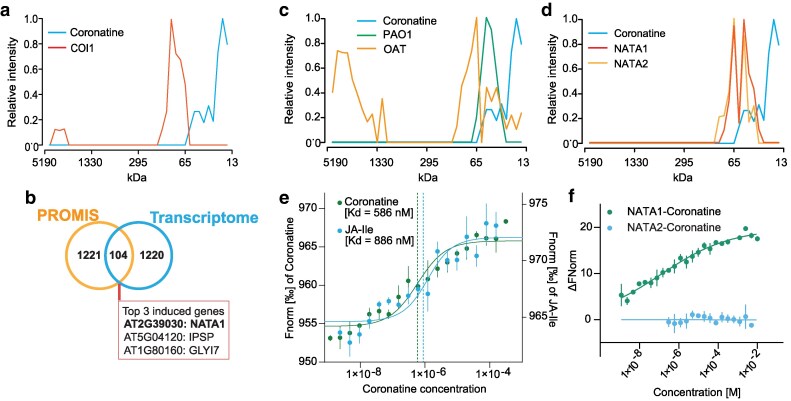
Coronatine binding to NATA1. a and c to d) Co-elution profile of coronatine with selected proteins specifically in the infection PROMIS separation. Several co-eluting proteins involved in polyamine biosynthesis are annotated. b) Candidate selection for coronatine-binding proteins. Venn diagram showing overlap between coronatine co-fractionating proteins from PROMIS (PCC > 0.9) and coronatine-regulated genes (Hayashi et al. 2023; FC > 2, adjusted *P* < 0.05). e) MST assay showing binding between NATA1 and coronatine and JA-Ile. The Kd was determined to be 586 and 889 nm, respectively. f) MST comparison between NATA1–coronatine and NATA2–coronatine. No detectable binding of coronatine to NATA2 was observed under identical MST conditions. All MST and activity assays were performed in at least 3 independent titrations. The error bar represents ± SEM.

As described above, a primary challenge in any CF-MS workflow, including PROMIS, is distinguishing true PMIs from coincidental co-elution (Schlossarek et al. 2023). One metabolite that stood out in our infection dataset was coronatine, a known *Pto* DC3000-derived virulence factor. Coronatine was detected exclusively in infected samples and was present in protein-containing fractions (Fig. 4a). However, its elution profile did not overlap with that of the canonical receptor COI1 (Katsir et al. 2008) (Fig. 4a). COI1 eluted near its expected monomeric molecular-weight range, and JAZ, the interacting proteins of COI1, were not detected in our proteomics dataset. These observations motivated us to search for additional coronatine-associated host proteins.

Coronatine co-eluted with a subset of host proteins across an apparent molecular-weight range of approximately 15 to 65 kDa, displaying 3 distinct elution maxima (Fig. 4a). Even when applying a stringent PCC > 0.9 threshold to define proteins co-fractionating with coronatine, the resulting list comprised more than 1,000 candidates, necessitating additional criteria for prioritization. We therefore intersected the co-fractionating protein list with proteins whose expression is strongly induced by coronatine treatment (adjusted *P* < 0.05; fold change > 2) (Hayashi et al. 2023), yielding 104 proteins meeting both criteria (Fig. 4b). From this subset, we focused on enzymes linked to polyamine metabolism (Fig. 4c and d), and in particular on the *N*-acetyltransferase NATA1, which showed the strongest induction in response to coronatine based on fold change and has previously been implicated in polyamine-associated defense outputs during infection (Adio et al. 2011; Lou et al. 2016).

Consistent with this prioritization, NATA1 abundance was markedly elevated in infected samples compared to mock and SAR separations (Fig. S1). In the control separation, the dominant NATA1 elution peak corresponded to a fraction estimated at 65.09 kDa, close to the expected mass of a NATA1 dimer (≈52 kDa). In infected samples, NATA1 exhibited 2 elution maxima—one at 65.09 kDa and a second at 41.38 kDa—of which the latter co-eluted with coronatine (Fig. S1).

To test for direct binding, we performed microscale thermophoresis (MST) using recombinant NATA1 and coronatine. A clear interaction was detected, with a dissociation constant (Kd) of 586 nm (Fig. 4e). For comparison, jasmonoyl-isoleucine (JA-Ile), a structural mimic of coronatine, also bound NATA1 with comparable affinity (Kd = 889 nm) (Fig. 4e). To assess specificity, we tested NATA2, a homolog of NATA1 (78% identity) that also co-eluted with coronatine in PROMIS (Fig. 4d). However, NATA2 showed no detectable interaction with coronatine under any tested condition (Fig. 4f), supporting specificity of the NATA1–coronatine interaction.

### Coronatine disrupts NATA1 dimerization without inhibiting its catalytic activity

**Figure 5:**
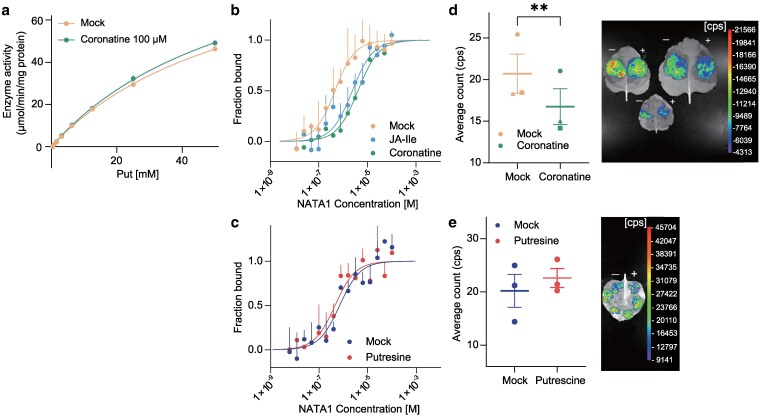
Coronatine-mediated disruption of NATA1 dimerization. a) *N-*acetyltransferase activity of NATA1 in the presence or absence of 100 *μ*m coronatine. b to c) MST-based quantification of NATA1 dimerization. Dimer formation occurred with a Kd of 412 nm in mock, but was reduced to 5-fold by coronatine and 10-fold by JA-Ile b) and showed slight increase by putrescine c). All MST and activity assays were performed in at least 3 independent titrations. The error bar represents ± SEM. d-e) Split-luciferase complementation assay in *N. benthamiana* showing decreased luminescence upon coronatine treatment d), but no change in putrescine treatment e). Points indicate individual replicates, and horizontal lines show mean ± SEM. Statistical significance was assessed using an unpaired *t-*test, *P* < 0.01.

We next investigated whether coronatine binding influences NATA1's enzymatic activity using an in vitro *N*-acetyltransferase assay with putrescine as the substrate. Surprisingly, coronatine treatment did not significantly alter NATA1 activity, with the exception of the lowest putrescine concentration (Fig. 5a; Fig. S2). This finding prompted us to explore an alternative hypothesis; rather, than inhibiting enzyme catalysis, coronatine may affect NATA1 dimerization. The hypothesis is supported by the co-fractionation data (Fig. S1). Coronatine co-elutes with the NATA1 peak corresponding to a monomeric form rather than a dimer, based on its estimated mass (Fig. S1). To test the impact of coronatine directly, we used MST to quantify NATA1–NATA1 dimer formation. In the absence of coronatine, NATA1 dimerized with a Kd of 412 nm (Fig. 5b). However, coronatine treatment reduced dimerization affinity by approximately 5-fold (Kd of 2.35 *μ*m), while JA-Ile reduced it by 10-fold (Kd of 4.01 *μ*m) (Fig. 5b). Conversely, putrescine, the well-known substrate of NATA1, had no significant effect on the dimerization affinity (Fig. 5c). To assess whether coronatine disrupts NATA1 dimerization in plants, we performed a split-luciferase complementation assay in *Nicotiana benthamiana*. Co-expression of NATA1 fusion constructs produced robust luminescence in control and putrescine-treated leaves (Fig. 5e), whereas treatment with coronatine (100 *μ*m) significantly reduced luciferase activity (Fig. 5d). Thus, across orthogonal assays, coronatine consistently depresses NATA1 dimer formation rather than inhibiting catalytic activity in vitro, supporting a structural mechanism of action.

### In silico docking and mutational validation of the NATA1–coronatine interface

**Figure 6:**
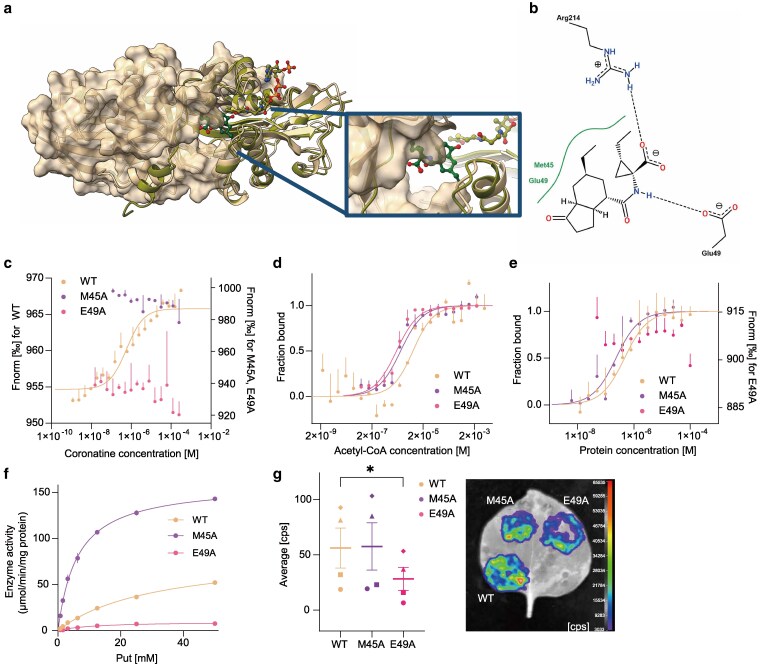
In silico docking and mutational validation of the NATA1–coronatine interface. a) Relaxed complex of NATA1, acetyl-coenzyme A, and coronatine aligned to the NATA1 dimer. Insert: the predicted binding pose of coronatine docked to the right monomer sterically clashes with the surface area of chain B. b) 2D interaction diagram of NATA1 and coronatine. The interaction appears mainly driven by hydrophobic interactions and hydrogen bonds between coronatine and amino acid residues 45, 49, and 214 of NATA1. c) MST assays showing that the M45A and E49A substitutions abolish coronatine binding relative to WT. d) MST assays with acetyl-CoA demonstrating increased cofactor affinity for M45A and E49A. e) MST-based quantification of NATA1 and its variants. f) *N*-acetyltransferase activity of NATA1 and its variants in the presence or absence of 100 *μ*m coronatine. All MST assays were performed in at least 3 independent titrations. Error bars represent ± SEM. g) Split-luciferase complementation assay in *N. benthamiana* showing NATA1 self-association for WT and interface variants. Points indicate individual replicates, and horizontal lines show mean ± SEM. Statistical significance was assessed using an unpaired *t-*test, *P* < 0.01. Left, quantification of luminescence signals (average counts per second, cps); right, representative luminescence images.

To further investigate coronatine binding, we performed in silico docking simulations using the AlphaFold-predicted monomer structure of NATA1 (Fig. 6a and b). The predicted structure of the NATA1 monomer in complex with acetyl-coenzyme A and coronatine is shown in Fig. 6a. Acetyl-coenzyme A is predicted to bind to its known binding site as annotated in UniProt Knowledgebase (UniProt Consortium 2025) with a *predicted template modeling* (pTM) score of 0.79. The high confidence of this prediction is captured by a chain pair *interface predicted template modeling* (ipTM) for NATA1 and acetyl-coenzyme A of 0.96. For the interaction with coronatine, an ipTM value of 0.72 was obtained. It should be noted that the AF-predicted NATA1 monomer is biased toward the dimeric state, because the C-terminal region (residues 202 to 228) retains a protruding conformation characteristic of the dimer, even when the second protomer is not present. As amino acid residues 211, 212, and 214 are part of the C-terminus and are found within a 4 Å radius of coronatine, this bias might influence the scoring of the NATA1 and coronatine interaction. This is reflected by the higher *predicted local distance difference test* (pLDDT) values around 70 for the cyclohexane and cyclopentane ring of coronatine as well. The pTM score for coronatine was 0.3. This is, at least partially, driven by the fact that the TM score is quite strict for small structures.

Aligning the complex of NATA1, acetyl-coenzyme A, and coronatine to chain A of the NATA1 dimer shows that the cyclopropane ring of coronatine and its attached ethyl group and nitrogen atom clash with the surface area of chain B of the NATA1 dimer (Fig. 6a). This supports the hypothesis that coronatine binding may potentially interfere with NATA1 dimerization. The interaction diagram obtained for the relaxed structure of the complex shows that the interaction of coronatine and NATA1 might be mainly driven by hydrophobic interactions and hydrogen bonds with amino acid residues 45, 49, and 214 as shown in Fig. 6b. Building on this model, we next asked whether the predicted contact residues are functionally sensitive for both coronatine binding and NATA1 dimerization.

Substitution of M45 or E49 fully abolished coronatine binding (Fig. 6c), demonstrating that the predicted binding surface is indeed functionally sensitive. Interestingly, these interface mutations produced divergent effects on NATA1 behavior beyond ligand recognition. While cofactor affinity showed a modest increase relative to WT (Fig. 6d), dimerization and catalytic activity were differentially rewired, with M45A showing a modest, nonsignificant trend toward increased dimerization affinity and elevating enzymatic activity (Fig. 6e and f), whereas E49A eliminated dimerization and abolished activity (Fig. 6e to g). In addition, coronatine no longer altered dimerization or enzymatic activity in the M45A variant (Fig. S3), further supporting that coronatine-dependent effect on NATA1 dimerization requires an intact interface. In contrast to M45A and E49A, mutating the third predicted contact site (R214A) did not simply eliminate coronatine binding, but instead produced a pronounced defect in acetyl-CoA binding and a near-complete loss of enzymatic activity (Fig. S4a, b, and e). We further tested R183, which has been reported to contribute to ligand binding by shaping the polyamine pocket similar to R214 (Hameed et al. 2024). While the R183A substitution had little to no effect on coronatine binding (Fig. S4c), it reduced acetyl-CoA affinity and decreased enzymatic activity to a level comparable to R214A (Fig. S4d and e).

Taken together, these results experimentally validate that all 3 residues highlighted by the in silico model contribute to NATA1–coronatine interaction. Moreover, the contrasting phenotypes of M45A and E49A indicate that altered dimerization can translate into substantial changes in enzymatic activity. Importantly, R214A and R183A results show that this relationship is not governed by dimerization alone but can also be modulated through changes in acetyl-CoA binding affinity, providing an additional route by which mutations at the ligand/substrate pocket impact enzymatic function.

## Discussion

### PROMIS as an interorganismal PMI discovery platform during infection

This study highlights an application of PROMIS in interorganismal research, demonstrating its capacity to resolve protein–metabolite co-fractionation patterns during pathogen infection. While PROMIS has previously been used to map protein–small molecule interactomes in diverse systems, including Arabidopsis, *Escherichia coli*, and yeast (Veyel et al. 2018; Luzarowski et al. 2021; Schlossarek et al. 2022; Wagner et al. 2025), its deployment here in native lysates from *Pto* DC3000-infected Arabidopsis tissue extends the approach to host–pathogen molecular crosstalk.

A key requirement for CF-MS-based PMI discovery is that native protein assemblies remain intact during fractionation. To address this, we benchmarked our protein elution profiles against curated Arabidopsis reference complexes derived from integration of CORUM and reference CF-MS datasets (Yang et al. 2025). Using an apparent multimerization ratio (Rapp), the large majority of benchmark proteins eluted in multimeric states and the Rapp values observed here correlated strongly with those from a reference Arabidopsis SEC-CF-MS study (Pearson *r* = 0.89) (Fig. 1d). However, at the PMI level, co-fractionation remains probabilistic. Correlation between elution profiles can arise from genuine biochemical association, indirect co-elution in larger assemblies, or chance of co-migration, particularly in fraction regions, where many proteins elute. To make this limitation explicit and quantitatively grounded, we examined PCC distributions globally and compared them to randomized controls. In our dataset, known metabolite–protein pairs curated from STITCH shift toward higher PCC values relative to all PMIs (Fig. 1h), and this enrichment is lost in randomized controls, supporting that PCC-based scoring captures true signal. Nevertheless, even with stringent PCC thresholds, large candidate sets remain, underscoring that PROMIS primarily serves as a discovery and prioritization framework, and that additional ranking criteria and independent validation are typically required to establish specific interactions.

### PROMIS reveals infection-associated remodeling of protein assemblies and metabolite-associated complexes

PROMIS datasets can be mined for infection-associated changes in protein–protein assemblies within the resolved molecular-weight range, providing an additional proof of concept for condition-dependent changes in complex behavior and composition. Here, we highlight several PPI examples for which our data indicate infection-dependent changes in apparent multimerization and where both partners co-fractionate in the infection and SAR datasets (Fig. 2b). Notably, the PSL4–PSL5 protein complex (Fig. 2b) has previously been implicated in plant defense responses. PSL4 and PSL5 comprise the β- and α-subunits of the endoplasmic reticulum–resident glucosidase II and function in *N*-glycan processing and protein quality control. This complex is required for stable accumulation and sustained signaling of the EF-Tu receptor EFR and has therefore been directly linked to Arabidopsis innate immune responses (Lu et al. 2009). A second example is N-terminal acetyltransferase B (NatB), composed of catalytic and regulatory subunits (Fig. 2b), which modifies a subset of newly synthesized proteins. Because N-terminal acetylation can influence protein half-life via the Ac/N-end rule pathway, NatB has been proposed to contribute to proteostasis-based regulation. Importantly in the context of immunity, NatB was shown to acetylate the Met2-initiated isoform of the NLR protein SNC1, promoting its stabilization and thereby helping to fine-tune NLR homeostasis and immune regulation (Xu et al. 2015). Less is known about the other 2 complexes highlighted here (Fig. 2b) in the context of plant immunity. However, both are established contributors to translational regulation. The tRNA guanine methyltransferase complex catalyzes conserved guanine methylation of tRNAs, a modification important for tRNA stability, translational fidelity, and efficient protein synthesis (Wang et al. 2017), whereas aspartyl-tRNA ligases 1 and 2 charge tRNA with aspartate and primarily function in core translational processes (Duchêne et al. 2005). Given the emerging links between translational control and immune outputs, infection-associated shifts in the co-fractionation behavior of these assemblies may reflect broader reprogramming of gene expression during defense.

In parallel to infection-associated remodeling of protein–protein complex, several metabolites were captured in protein-containing fractions, including pathogen-derived virulence factors (coronatine and phevamine A) and host defense-associated metabolites (eg camalexin and polyamines), and multiple features displayed clear condition-dependent shifts in elution behavior (Fig. 3a to d). Thymidine and serotonin provide illustrative examples whose profiles differed between mock and infection/SAR conditions, consistent with broader remodeling of metabolic and signaling states during immune activation. Likewise, sulforaphane and the dipeptide Ala–Ile exhibited elution patterns in infected samples that diverged from both mock and SAR, highlighting that local infection and systemic immunity can impose distinct reconfigurations on metabolite-associated assemblies.

Together, these examples illustrate that PROMIS captures infection- and SAR-associated shifts in both protein assemblies and metabolite distributions within protein-containing fractions, providing a resource for hypothesis generation. Establishing the underlying mechanisms and distinguishing direct physical interactions from co-migration will require targeted follow-up experiments and orthogonal validation.

### Coronatine engages NATA1 and remodels its assembly

A key finding of this work is the identification of NATA1 as a previously unrecognized coronatine-binding protein. Although COI1 is a well-established receptor for coronatine/JA-Ile (Katsir et al. 2008; Sheard et al. 2010), we did not observe coronatine–COI1 co-elution in our PROMIS profiles. One possible explanation is that coronatine recognition by COI1 is typically stabilized by formation of a higher-order complex with JAZ proteins (Sheard et al. 2010). In our dataset, JAZ proteins were not detected by proteomics, consistent with their low abundance or rapid turnover. In addition, ligand-triggered JAZ ubiquitination and degradation can further limit steady-state accumulation of the active COI1–JAZ complex. Thus, the absence of COI1–coronatine co-fractionation in our experimental context may reflect limitations of detecting transient or low abundance complexes in native lysates rather than a lack of coronatine binding to COI1 in vivo. While COI1 is a well-established receptor for coronatine and its structural analog JA-Ile (Yan et al. 2009), our PROMIS data indicated that coronatine co-elutes with a distinct subset of host proteins, including enzymes involved in polyamine metabolism. Among these, NATA1 stood out due to its infection-specific expression profile and its known role in modulating polyamine levels (Adio et al. 2011; Lou et al. 2016). NATA1 functions as an *N*-acetyltransferase catalyzing the acetylation of putrescine and its precursor ornithine (Adio et al. 2011; Lou et al. 2016), thereby contributing to polyamine homeostasis and redox balance. Because polyamine biosynthesis is upregulated during pathogen infection and putrescine serves as a precursor for spermidine and spermine, NATA1-mediated acetylation can divert putrescine from polyamine synthesis and dampen antimicrobial defense. Consistent with this, NATA1 expression is induced by JA and coronatine, and *nata1* mutants show reduced acetylation, elevated polyamine levels, increased H_2_O_2_ production, and enhanced defense gene expression, supporting a role for coronatine-induced putrescine acetylation in suppressing immunity (Adio et al. 2011; Lou et al. 2016).

In our experiments, we confirmed a direct interaction between NATA1 and coronatine without a measurable effect on catalytic turnover under our assay conditions. By contrast, coronatine disrupted NATA1 dimerization both in vitro and in planta, and in silico docking further supported a model in which coronatine binding is structurally incompatible with the dimer interface (Fig. 6a and b). The mutational interrogation guided by the docking-predicted interface showed that all 3 predicted contact sites contribute to coronatine binding in vitro. Notably, substitutions at E49 revealed that perturbations at the predicted interface can strongly impact NATA1 dimerization, significantly reducing enzyme activity (Fig. 6e and f).

Interestingly, mutation at the third predicted site produced a different phenotype from M45A/E49A. R214A had comparatively modest effects on coronatine binding but severely impaired acetyl-CoA binding and strongly reduced enzymatic activity (Fig. S4). Moreover, we tested R183 because, although it was not prioritized by our docking model Hameed et al. (2024) reported that R183 together with R214 forms the back of the polyamine pocket and is critical for substrate binding to NATA1. While R183A had little to no effect on coronatine binding, it reduced acetyl-CoA affinity and decreased enzyme activity to a degree similar to R214A (Fig. S4), consistent with functional coupling between “substrate-side” architecture and cofactor engagement. Mechanistically, this is in line with structural principles established for the GCN5-related *N*-acetyltransferase (GNAT) superfamily and spermidine/spermine *N*1-acetyltransferase (SSAT), where the acetyl-CoA pantetheine arm is positioned adjacent to the amine-accepting pocket and active sites commonly place positively charged residues at the back of the polyamine pocket to orient the amine for acetyl transfer (Montemayor and Hoffman 2008; Filippova et al. 2011). Together, these results support a model in which coronatine engages a defined surface interface on NATA1 that is mechanistically linked to both dimerization and cofactor/substrate pocket architecture, explaining how coronatine can reproducibly perturb NATA1 assembly.

What, then, is the biological role of coronatine binding to NATA1? One apparent discrepancy in our in vitro data is that coronatine consistently reduced NATA1 dimerization yet did not produce a pronounced decrease in catalytic turnover under the enzyme-assay conditions used here. Importantly, the M45A and E49A variants provide experimental support that perturbations at the predicted interface can couple dimerization state to enzymatic output, with loss of dimerization coinciding with loss of activity. We therefore consider the possibility that the activity assay, as implemented, has limited sensitivity to detect modest decreases in turnover caused by partial dimer destabilization. Specifically, since we used substrate for the enzymatic assay in 1 to 50 mm range, conditions under which substrate-rich turnover can buffer modest reductions in the effective pool of active dimers. Consistent with partial masking, at the lowest substrate concentration that remained quantifiable by our assay (625 *µ*m), coronatine produced ∼57% reduction in activity (Fig. S2). However, because substrate concentrations could not be reduced further without falling below the detection limit, we cannot exclude that the functional consequence of dimer destabilization is more evident under substrate-limiting conditions.

## Conclusion

In conclusion, we applied PROMIS to native lysates from *Pto* DC3000-infected Arabidopsis. Across mock, infected, and SAR tissues, PROMIS captured widespread infection-associated remodeling of protein assemblies and metabolite distributions within protein-containing fractions, providing a resource for hypothesis generation and targeted follow-up. Using a prioritization strategy, we identified NATA1 as a coronatine-binding protein and validated this interaction biochemically. While coronatine did not elicit a strong decrease in NATA1 turnover under the assay conditions used here, docking- and mutagenesis-based analyses supported a functional coupling between NATA1 self-association and catalytic activity. Together, these results illustrate how native interactome profiling can uncover infection-responsive regulatory nodes and suggest that coronatine may modulate host polyamine acetylation through remodeling of NATA1 assembly.

## Materials and methods

### Plant growth

*A. thaliana* (Col-0) plants were grown under short-day conditions (12-h light/12-h dark) at 20/18 °C, 60% relative humidity, and a light intensity of 150 *μ*mol m^−2^ s^−1^. Plants aged 5 to 6 wk were used for experiments. *N. benthamiana* plants were grown under long-day conditions (16-h light/8-h dark) at 25 °C, 60% humidity, and 125 *μ*mol m^−2^ s^−1^ irradiance for 4 wk.

### Bacterial growth

*Pto* DC3000 was streaked onto King's B agar plates supplemented with 100 *µ*g/mL rifampicin and incubated at 28 °C for 24 to 48 h. A single colony was used to inoculate liquid KB medium containing rifampicin and cultured at 28 °C with shaking (120 to 140 rpm). Once the culture reached OD_600nm_ of 0.6 to 0.8, cells were harvested by centrifugation (2,500 × *g*, 10 min), washed, and resuspended in sterile water to OD_600nm_ = 0.2 for use as the inoculum. For recombinant protein production, *E. coli* BL21 (DE3) strains expressing His-tagged NATA1, its variants, or NATA2 were cultured in LB overnight at 37 °C. A 1% inoculum was transferred to terrific broth autoinduction medium (0.5 g/L glucose, 2 g/L lactose, 12 g/L tryptone, 24 g/L yeast extract, 6.8 g/L KH_2_PO_4_, 7.1 g/L Na_2_HPO_4_) and incubated at 37 °C for 4 h, followed by overnight culture at 16 °C.

### Plant infection

Arabidopsis plants were infiltrated with *Pto* DC3000 using a syringe without a needle, following a modified protocol from Katagiri et al. (2002). Bacterial suspensions were gently pressed against the abaxial side of leaves to introduce the inoculum. After infiltration, leaves were rinsed with tap water, and plants were maintained under dim light. Leaf samples were frozen in liquid nitrogen and stored at −80 °C (Katagiri et al. 2002).

### PROMIS workflow

Frozen leaf material was ground in ice-cold lysis buffer (50 mm AmBIC, 150 mm NaCl, 1.5 mm MgCl_2_, 5 mm DTT, 1 mm PMSF, 1 × cOmplete EDTA-free protease inhibitor cocktail, 0.1 mm Na_3_VO_4_, 1 mm NaF). Lysates were cleared by centrifugation (4,000 × *g*, 10 min, 4 °C), followed by ultracentrifugation (35,000 rpm, 1 h, 4 °C) to obtain the soluble fraction. PROMIS separations were performed as previously described (Sokolowska et al. 2019). The soluble fraction (40 mg of protein) was filtered (Amicon Ultra-15, 10 kDa MWCO) and loaded onto a Sepax SRT SEC-300 column (21.2 × 300 mm) (Sepax Technologies, Inc., Delaware Technology Park, separation range 1.2 MDa to 10 kDa) connected to an ÄKTA explorer 10 system (GE Healthcare Life Sciences). Separation was performed at 4 °C and 7 mL/min using buffer (50 mm AmBIC pH 7.5, 150 mm NaCl, 1.5 mm MgCl_2_). Fractions (1 mL) were collected from the 39 to 84 mL elution volume, snap frozen, lyophilized, and stored at −80 °C. For protein and metabolite extraction, lyophilized fractions, starting with fraction A6, were extracted using a methyl-tert-butyl ether (MTBE)/methanol/water solvent system as adapted from Sokolowska et al. (2019) Protein pellets and polar fractions were dried and stored at −80 °C for further analysis.

### Proteomics

Protein quantification was performed using the Bradford assay. Pellets were solubilized in 30 *µ*L urea buffer (6 m urea, 2 m thiourea in 40 mm AmBIC). A total of 20 *µ*g of proteins were reduced, alkylated, and digested using LysC/Trypsin (Promega Corp., Fitchburg, WI) according to the manufacturer's instructions. Desalting was performed using C18 Empore extraction discs (3M). Proteins were concentrated using the centrifugal evaporator and stored at −80 °C until measured. Dried peptides were resuspended in 60 *µ*L loading buffer (2% ACN, 0.2% TFA), and 3 *µ*L (equivalent to 0.8 to 1.0 *µ*g of peptides) was injected onto a C18 reversed-phase column connected to an ACQUITY UPLC M-Class system with a 120 min gradient. The gradient started from 3.2% and increased to 7.2% ACN in 20 min, to 24.8% ACN over 70 min, and to 35.2% ACN over 30 min, followed by a 5 min washout with 76% ACN. The Thermo Q Exactive HF operated with a data-dependent method as follows: MS full scans were performed in FTMS with resolution set to 120,000, from 300.0 to 1,600.0 m/z, a maximum fill time of 50 ms, and an AGC target value of 3xe^6^ ions. A maximum of 12 data-dependent MS^2^ scans was performed in the ion trap set to an AGC target of 1xe^5^ ions with a maximal injection time of 100 ms. Precursor ion fragmentation was achieved with collision-induced fragmentation with a normalized collision energy of 27 and an isolation width of 1.2 m/z. Charge states of 1 and ≥7 were excluded. Raw proteomics files were analyzed using MaxQuant software with Andromeda, an integrated peptide search engine (Cox and Mann 2008) with the following settings: maximum of 2 missed cleavages were allowed, and threshold for peptide validation was set to 0.01 using a decoy database methionine oxidation, and N-terminal acetylation was considered as variable modification, while cysteine carbamidomethylation as a fixed modification. The minimum length of peptide was set to at least 7 amino acids. Moreover, the following options were selected: “label-free quantification” and “match between runs.” Peptides were identified for Arabidopsis datasets using: *A. thaliana* UniProt protein sequences.

### Metabolomics

After extraction, the dried aqueous metabolites were measured using ultraperformance liquid chromatography (UPLC) coupled with a Q Exactive mass spectrometer (Thermo Fisher Scientific) in positive and negative ionization modes, as described earlier (Veyel et al. 2018). Expressionist Refiner MS 12.0 (Genedata AG, Basel, Switzerland) was used for processing the LC–MS data. In-house library of authentic reference compounds was used to identify molecular features allowing 10 ppm mass deviation and dynamic retention time deviation (maximum 0.2 min).

### Data analysis

Elution profiles were analyzed using standard settings in the PROMIsed app (Schlossarek et al. 2021), including preprocessing, deconvolution, and integration steps. We removed all protein and metabolite maxima that peaked in fraction A6 corresponding to the first analyzed fraction. To calculate the PCC, we used preprocessed and deconvoluted elution profiles, and the N/A values were substituted by 0 (Schlossarek et al. 2021). To assess whether our dataset recapitulates known interactions, we compiled a reference set from STITCH (Kuhn et al. 2010) and retained edges with experimental evidence score ≥ 0.4.

### Protein purification

Frozen bacterial cells from terrific broth autoinduction were used for subsequent protein purification. The soluble fraction was purified using a 1 mL HisTrap (Cytiva) column connected to NGC Quest 10 (Bio-Rad), previously equilibrated with the buffer containing 25 mm Tris–HCl pH 8.0, 300 mm NaCl, 5% (v/v) glycerol, 10 mm imidazole. The column was washed with 10 mL of the buffer, and the recombinant protein was eluted with a linear gradient of imidazole (10 to 300 mm). The fractions containing the protein of interest were collected and concentrated and desalted using ultracentrifugal filters. The purified protein was stored in 0.1 m Tris–HCl buffer pH 7.5 at 4 °C for use in future experiments.

### Site-directed mutagenesis

Site-directed mutagenesis of NATA1 gene was performed using Q5 site-directed mutagenesis kit (New England Biolabs) according to the manufacturer's protocol. Mutagenic primers were designed using NEBasechanger online tool (NEB) and are listed in Table S1. The plasmid pET28a-NATA1 served as the template for PCR amplification. Following sequence verification of the resulting mutant constructs, recombinant protein expression and purification were carried out under the same conditions as those described above.

### Microscale thermophoresis

Ligands (coronatine, JA-Ile, and acetyl-CoA) were serially diluted in MST buffer (50 mm potassium phosphate, pH 6.5; 7.6 mm (NH_4_)_2_SO_4_; 1.7 mm MgCl_2_; 1.7 mm NaCl; 0.05% Tween-20) using a 16-step, 2-fold dilution series. RED-labeled His-tagged NATA1 and its variants (625 nm) were mixed with each ligand (6 *µ*L per sample) and loaded into premium capillaries (NanoTemper Technologies). Measurements were performed on a Monolith instrument (NanoTemper Technologies). Data were analyzed using standard MST traces, and normalized fluorescence (Fnorm) was plotted against ligand concentration to calculate Kds. To enable cross-experiment comparison, fraction-bound values were computed to minimize dependence on amplitude and initial fluorescence. Data analysis was conducted using Monolith Analysis software. For dimerization assays, NATA1 and its variants were labeled with RED-NHS dye (NanoTemper Technologies) following the manufacturer's protocol. Labeled NATA1s (50 nm) were incubated with unlabeled NATA1s (starting at 200 *µ*m) in the presence or absence of 100 *µ*m coronatine, 100 *µ*m JA-Ile, or 500 *µ*m putrescine. Each experiment was performed in triplicate.

### Enzymatic assays

NATA1 enzymatic activity was measured using a Multiskan Skyhigh Microplate Spectrophotometer (Thermo Fischer Scientific). The reaction mixture contained 50 mm Tris–HCl (pH 6.8), 20 mm NaCl, 1 mm acetyl-CoA, 2 *μ*m purified NATA1 or variant protein, and varying concentrations of putrescine as the substrate. To assess the effect of coronatine on enzymatic activity, 100 *μ*m coronatine was included in the reaction mixture. Reactions were incubated at 30 °C for 10 min and terminated by the addition of an equal volume of quenching buffer consisting of 6 m guanidine hydrochloride and 0.1 m Tris–HCl (pH 8.0). The production of free coenzyme A was quantified by adding Ellman's reagent [0.2 mm 5,5′-dithiobis-(2-nitrobenzoic acid) (DTNB), 0.1 M Tris–HCl (pH 8.0), and 1 mm EDTA] and measuring the absorbance at 412 nm. All measurements were performed in technical triplicates, and the data were plotted using GraphPad Prism 10 (GraphPad software).

### Split-luciferase complementation assay

The split-luciferase complementation assay was performed as previously described (Wang et al. 2021). Briefly, the coding sequences of NATA1 and its variants were amplified from pET28a-NATA1, using gene-specific primers listed in Table S1. PCR products were cloned into pENTR/D-TOPO (Thermo Fischer Scientific) to generate Gateway-compatible entry clones. The resulting entry constructs were recombined into pGWB-NLuc and pGWB-CLuc destination vectors via LR Clonase II reaction (Thermo Fischer Scientific), according to the manufacturer's instructions [pGWB-nLUC was a gift from Alberto Macho (Addgene plasmid # 174050) (Yu et al. 2020)]. Constructs were introduced into *Agrobacterium tumefaciens* by chemical transformation, using competent cells prepared as described previously (Wang et al. 2021). Transformed *A. tumefaciens* cultures were grown overnight at 28 °C until reaching an OD_600_ of 1.0. Cells were harvested by centrifugation at 4,000 × *g* for 10 min, and the pellets were resuspended in infiltration buffer containing 10 mm MES (pH 5.7), 10 mm MgCl_2_, and 150 *μ*m acetosyringone to an OD_600_ of 1.0. Equal volumes of the bacterial suspensions carrying NLuc- and CLuc-fusion constructs were mixed and incubated in the dark at room temperature for 3 h. The mixtures were then infiltrated into the abaxial side of 4-wk-old *N. benthamiana* leaves using a needleless syringe. Infiltrated plants were incubated in a growth chamber for 28 h. To test the effect of different treatments, leaves were infiltrated with 0.02% DMSO (control), 100 *μ*m coronatine, or 500 *μ*m putrescine. After leaves were fully dried, 1 mm luciferin was infiltrated, and plants were incubated for 10 min in the dark to minimize background chlorophyll luminescence. Experiments were repeated 3 times in triplicate. Bioluminescence signals were captured using a CCD imaging system (NightShade LB 985, Berthold Technologies), and image analysis was conducted as described in Wang et al. (2021). To compare the expression level of NATA1 split-luciferase fusion variants, *N. benthamiana* leaves transiently expressing NATA1 split-luciferase fusion constructs (WT, M45A, E49A) were harvested at the same time point as used for the split-luciferase assay. Total proteins were extracted in lysis buffer (20 mm Tris–HCl, pH 8.0, 150 mm NaCl, 1 mm EDTA, 1% of Triton X-100, 0.1% of SDS, 10 mm DTT, 1× protease inhibitor cocktail, 1× SDS sample buffer), separated by SDS–PAGE, and transferred to a PVDF membrane. nLUC- and cLUC-fusion proteins were detected by immunoblotting using an antiluciferase antibody (Sigma, L0159) (Fig. S5).

### Structural prediction

All protein structures were predicted using an in-house installation of AlphaFold (AF) 3 (Abramson et al. 2024). Each structure prediction was initialized using 5 random seeds. Since the N-terminus of NATA1 (UniProt ID: Q9ZV05) including residues 1 to 20 was predicted to be disordered for the monomer (Varadi et al. 2024), these residues were excluded for the prediction of the homodimer as well as prediction of the complex with acetyl-coenzyme A (CCD: ACO) and coronatine (CCD: OGK). All predicted structures were evaluated based on the internal metrics of AF, including ipTM, pTM, pLDDT, and PAE. For all predictions, the best ranking model was optimized using the relax protocol as implemented in Rosetta (Leaver-Fay et al. 2011) with 5 cycles of relaxation, which includes alternating side-chain optimization and energy minimization. Protein visualizations were created using ChimeraX v1.7.(Zhou et al. 2023). For clarity of the visualizations, interactions determined by DDMut were remodeled in ChimeraX. The 2D interaction diagram for NATA1 and coronatine was obtained using the ProteinsPlus webserver (Ehrt et al. 2025).

### Accession numbers

Sequence data from this article can be found in the GenBank/EMBL data libraries under accession numbers NATA1 (AT2G39030), NATA2 (AT2G39020), and COI1 (AT2G39940).

## Supplementary Material

kiag349_Supplementary_Data

## Data Availability

The PROMISed app is available at https://github.com/DennisSchlossarek/PROMISed. Proteomics and metabolomics data are available in supplementary materials. Proteomics data is available via ProteomeXchange (Deutsch et al. 2023) (Identifier: PXD069625). Metabolomics data is available *via* MassIVE repository (Identifier: MSV000100226).

## References

[kiag349-B1] Abramson J et al 2024. Addendum: accurate structure prediction of biomolecular interactions with AlphaFold 3. Nature. 636:E4. 10.1038/s41586-024-08416-7. 39604737 PMC11634763

[kiag349-B2] Adio AM et al 2011. Biosynthesis and defensive function of Nδ-acetylornithine, a jasmonate-induced Arabidopsis metabolite. Plant Cell. 23:3303–3318. 10.1105/tpc.111.088989. 21917546 PMC3203426

[kiag349-B3] Aryal UK et al 2014. A proteomic strategy for global analysis of plant protein complexes. Plant Cell. 26:3867–3882. 10.1105/tpc.114.127563. 25293756 PMC4247564

[kiag349-B4] Cai J, Aharoni A. 2022. Amino acids and their derivatives mediating defense priming and growth tradeoff. Curr Opin Plant Biol. 69:102288. 10.1016/j.pbi.2022.102288. 35987012

[kiag349-B5] Chodasiewicz M et al 2022. A novel role of 3,5-cAMP in the regulation of actin cytoskeleton in Arabidopsis [preprint]. bioRxiv 478439. 10.1101/2022.01.31.478439.

[kiag349-B6] Cox M, Mann M. 2008. MaxQuant enables high peptide identification rates, individualized ppb-range mass accuracies and proteome-wide protein quantification. Nat Biotechnol. 26:1367–1372. 10.1038/nbt.1511. 19029910

[kiag349-B7] Deutsch EW et al 2023. The ProteomeXchange consortium at 10 years: 2023 update. Nucleic Acids Res. 51:D1539–D1548. 10.1093/nar/gkac1040. 36370099 PMC9825490

[kiag349-B8] Duchêne A-M et al 2005. Dual targeting is the rule for organellar aminoacyl-tRNA synthetases in Arabidopsis thaliana. Proc Natl Acad Sci U S A. 102:16484–16489. 10.1073/pnas.0504682102. 16251277 PMC1283425

[kiag349-B9] Ehrt C et al 2025. ProteinsPlus: a publicly available resource for protein structure mining. Nucleic Acids Res. 53:W478–W484. 10.1093/nar/gkaf377. 40326518 PMC12230695

[kiag349-B10] Filippova EV et al 2011. Crystal structure of the novel PaiA N-acetyltransferase from Thermoplasma acidophilum involved in the negative control of sporulation and degradative enzyme production. Proteins. 79:2566–2577. 10.1002/prot.23062. 21633970 PMC3690761

[kiag349-B11] Gasperini D, Howe GA. 2024. Phytohormones in a universe of regulatory metabolites: lessons from jasmonate. Plant Physiol. 195:135–154. 10.1093/plphys/kiae045. 38290050 PMC11060663

[kiag349-B12] Gilbert M, Schulze WX. 2019. Global identification of protein complexes within the membrane proteome of Arabidopsis roots using a SEC-MS approach. J Proteome Res. 18:107–119. 10.1021/acs.jproteome.8b00382. 30370772

[kiag349-B13] Goel RK, Bithi N, Emili A. 2024. Trends in co-fractionation mass spectrometry: a new gold-standard in global protein interaction network discovery. Curr Opin Struct Biol. 88:102880. 10.1016/j.sbi.2024.102880. 38996623

[kiag349-B14] Hameed UFS et al 2024. Regulation of Arabidopsis polyamine acetylation by NATA1 and NATA2 [preprint]. bioRxiv 583282. 10.1101/2024.03.04.583282.

[kiag349-B15] Hayashi K et al 2023. Subtype-selective agonists of plant hormone co-receptor COI1-JAZs identified from the stereoisomers of coronatine. Commun Biol. 6:320. 10.1038/s42003-023-04709-1. 36966228 PMC10039919

[kiag349-B16] Katagiri F, Thilmony R, He SY. 2002. The Arabidopsis thaliana-pseudomonas syringae interaction. Arabidopsis Book. 1:e0039. 10.1199/tab.0039. 22303207 PMC3243347

[kiag349-B17] Katsir L, Schilmiller AL, Staswick PE, He SY, Howe GA. 2008. COI1 is a critical component of a receptor for jasmonate and the bacterial virulence factor coronatine. Proc Natl Acad Sci U S A. 105:7100–7105. 10.1073/pnas.0802332105. 18458331 PMC2383947

[kiag349-B18] Ketehouli T et al 2025. Secondary metabolites in plant-microbe interactions. J Appl Microbiol. 136:lxaf124. 10.1093/jambio/lxaf124. 40408276

[kiag349-B19] Kosmacz M et al 2018. Interaction of 2′,3′-cAMP with Rbp47b plays a role in stress granule formation. Plant Physiol. 177:411–421. 10.1104/pp.18.00285. 29618637 PMC5933139

[kiag349-B20] Kosmacz M, Sokołowska EM, Bouzaa S, Skirycz A. 2020. Towards a functional understanding of the plant metabolome. Curr Opin Plant Biol. 55:47–51. 10.1016/j.pbi.2020.02.005. 32224339

[kiag349-B21] Kuhn M et al 2010. STITCH 2: an interaction network database for small molecules and proteins. Nucleic Acids Res. 38:D552–D556. 10.1093/nar/gkp937. 19897548 PMC2808890

[kiag349-B22] Leaver-Fay A et al 2011. ROSETTA3: an object-oriented software suite for the simulation and design of macromolecules. Methods Enzymol. 487:545–574. 10.1016/B978-0-12-381270-4.00019-6. 21187238 PMC4083816

[kiag349-B23] Lee Y, Okita TW, Szymanski DB. 2021. A co-fractionation mass spectrometry-based prediction of protein complex assemblies in the developing rice aleurone-subaleurone. Plant Cell. 33:2965–2980. 10.1093/plcell/koab182. 34270775 PMC8462808

[kiag349-B24] Lee Y, Park HL, Yoon GM, Szymanski DB. 2025. Rapid ethylene-triggered protein complex remodeling in dark-grown Arabidopsis hypocotyls. Plant Physiol. 199:kiaf572. 10.1093/plphys/kiaf572. 41396865

[kiag349-B25] Lee Y, Szymanski DB. 2021. Multimerization variants as potential drivers of neofunctionalization. Sci Adv. 7:eabf0984. 10.1126/sciadv.abf0984. 33771868 PMC7997512

[kiag349-B26] Li Y et al 2021. Coupling proteomics and metabolomics for the unsupervised identification of protein–metabolite interactions in Chaetomium thermophilum. PLoS One. 16:e0254429. 10.1371/journal.pone.0254429. 34242379 PMC8270407

[kiag349-B27] Lou Y-R, Bor M, Yan J, Preuss AS, Jander G. 2016. Arabidopsis NATA1 acetylates putrescine and decreases defense-related hydrogen peroxide accumulation. Plant Physiol. 171:1443–1455. 10.1104/pp.16.00446. 27208290 PMC4902623

[kiag349-B28] Lu X et al 2009. Uncoupling of sustained MAMP receptor signaling from early outputs in an Arabidopsis endoplasmic reticulum glucosidase II allele. Proc Natl Acad Sci U S A. 106:22522–22527. 10.1073/pnas.0907711106. 20007779 PMC2799704

[kiag349-B29] Luzarowski M et al 2021. Global mapping of protein–metabolite interactions in Saccharomyces cerevisiae reveals that Ser-Leu dipeptide regulates phosphoglycerate kinase activity. Commun Biol. 4:181. 10.1038/s42003-021-01684-3. 33568709 PMC7876005

[kiag349-B30] McWhite CD et al 2020. A pan-plant protein complex map reveals deep conservation and novel assemblies. Cell. 181:460–474.e14. 10.1016/j.cell.2020.02.049. 32191846 PMC7297045

[kiag349-B31] Monte I . 2023. Jasmonates and salicylic acid: evolution of defense hormones in land plants. Curr Opin Plant Biol. 76:102470. 10.1016/j.pbi.2023.102470. 37801737

[kiag349-B32] Montemayor EJ, Hoffman DW. 2008. The crystal structure of spermidine/spermine N1-acetyltransferase in complex with spermine provides insights into substrate binding and catalysis. Biochemistry. 47:9145–9153. 10.1021/bi8009357. 18690703

[kiag349-B33] Moreno JC et al 2021. Tyr-Asp inhibition of glyceraldehyde 3-phosphate dehydrogenase affects plant redox metabolism. EMBO J. 40:e106800. 10.15252/embj.2020106800. 34156108 PMC8327957

[kiag349-B34] Olinares PDB, Ponnala L, van Wijk KJ. 2010. Megadalton complexes in the chloroplast stroma of Arabidopsis thaliana characterized by size exclusion chromatography, mass spectrometry, and hierarchical clustering. Mol Cell Proteomics. 9:1594–1615. 10.1074/mcp.M000038-MCP201. 20423899 PMC2938090

[kiag349-B35] O’Neill EM et al 2018. Phevamine A, a small molecule that suppresses plant immune responses. Proc Natl Acad Sci U S A. 115:E9514–E9522. 10.1073/pnas.1803779115. 30237288 PMC6187163

[kiag349-B36] Pang Z et al 2021. Linking plant secondary metabolites and plant microbiomes: a review. Front Plant Sci. 12:621276. 10.3389/fpls.2021.621276. 33737943 PMC7961088

[kiag349-B37] Piasecka A, Jedrzejczak-Rey N, Bednarek P. 2015. Secondary metabolites in plant innate immunity: conserved function of divergent chemicals. New Phytol. 206:948–964. 10.1111/nph.13325. 25659829

[kiag349-B38] Rojas CM, Senthil-Kumar M, Tzin V, Mysore KS. 2014. Regulation of primary plant metabolism during plant-pathogen interactions and its contribution to plant defense. Front Plant Sci. 5:17. 10.3389/fpls.2014.00017. 24575102 PMC3919437

[kiag349-B39] Schlossarek D et al 2021. PROMISed: a novel web-based tool to facilitate analysis and visualization of the molecular interaction networks from co-fractionation mass spectrometry (CF-MS) experiments. Comput Struct Biotechnol J. 19:5117–5125. 10.1016/j.csbj.2021.08.042. 34589187 PMC8453180

[kiag349-B40] Schlossarek D et al 2022. Rewiring of the protein-protein-metabolite interactome during the diauxic shift in yeast. Cell Mol Life Sci. 79:550. 10.1007/s00018-022-04569-8. 36242648 PMC9569316

[kiag349-B41] Schlossarek D et al 2023. Don’t let go: co-fractionation mass spectrometry for untargeted mapping of protein-metabolite interactomes. Plant J. 113:904–914. 10.1111/tpj.16084. 36575913

[kiag349-B42] Sheard LB et al 2010. Jasmonate perception by inositol-phosphate-potentiated COI1–JAZ co-receptor. Nature. 468:400–405. 10.1038/nature09430. 20927106 PMC2988090

[kiag349-B43] Sokolowska EM, Schlossarek D, Luzarowski M, Skirycz A. 2019. PROMIS: global analysis of PROtein-metabolite interactions. Curr Protoc Plant Biol. 4:e20101. 10.1002/cppb.20101. 31750999

[kiag349-B44] UniProt Consortium . 2025. UniProt: the universal protein knowledgebase in 2025. Nucleic Acids Res. 53:D609–D617. 10.1093/nar/gkae1010. 39552041 PMC11701636

[kiag349-B45] Varadi M et al 2024. AlphaFold protein structure database in 2024: providing structure coverage for over 214 million protein sequences. Nucleic Acids Res. 52:D368–D375. 10.1093/nar/gkad1011. 37933859 PMC10767828

[kiag349-B46] Venegas-Molina J, Molina-Hidalgo FJ, Clicque E, Goossens A. 2021. Why and how to dig into plant metabolite-protein interactions. Trends Plant Sci. 26:472–483. 10.1016/j.tplants.2020.12.008. 33478816

[kiag349-B47] Veyel D et al 2017. System-wide detection of protein-small molecule complexes suggests extensive metabolite regulation in plants. Sci Rep. 7:42387. 10.1038/srep42387. 28205532 PMC5304321

[kiag349-B48] Veyel D et al 2018. PROMIS, global analysis of PROtein–metabolite interactions using size separation in Arabidopsis thaliana. J Biol Chem. 293:12440–12453. 10.1074/jbc.RA118.003351. 29853640 PMC6093232

[kiag349-B49] Wagner M et al 2025. Mapping protein-metabolite interactions in *E. coli* by integrating chromatographic techniques and co-fractionation mass spectrometry. iScience. 28:112611. 10.1016/j.isci.2025.112611. 40491478 PMC12148380

[kiag349-B50] Wagner M, Zhang B, Tauffenberger A, Schroeder FC, Skirycz A. 2021. Experimental methods for dissecting the terra-incognita of protein-metabolite interactomes. Curr Opin Syst Biol. 28:100403. 10.1016/j.coisb.2021.100403.

[kiag349-B51] Walther D . 2023. Specifics of metabolite-protein interactions and their computational analysis and prediction. Methods Mol Biol. 2554:179–197. 10.1007/978-1-0716-2624-5_12. 36178627

[kiag349-B52] Wang L, Yu G, Macho AP, Lozano-Durán R. 2021. Split-luciferase complementation imaging assay to study protein-protein interactions in. Bio Protoc. 11:e4237. 10.21769/BioProtoc.4237. PMC8678545 35005082

[kiag349-B53] Wang Y et al 2017. Identification of tRNA nucleoside modification genes critical for stress response and development in rice and Arabidopsis. BMC Plant Biol. 17:261. 10.1186/s12870-017-1206-0. 29268705 PMC5740945

[kiag349-B54] Wang Y, Pruitt RN, Nürnberger T, Wang Y. 2022. Evasion of plant immunity by microbial pathogens. Nat Rev Microbiol. 20:449–464. 10.1038/s41579-022-00710-3. 35296800

[kiag349-B55] Xin X-F, He SY. 2013. Pseudomonas syringae pv. tomato DC3000: a model pathogen for probing disease susceptibility and hormone signaling in plants. Annu Rev Phytopathol. 51:473–498. 10.1146/annurev-phyto-082712-102321. 23725467

[kiag349-B56] Xu F et al 2015. Two N-terminal acetyltransferases antagonistically regulate the stability of a nod-like receptor in Arabidopsis. Plant Cell. 27:1547–1562. 10.1105/tpc.15.00173. 25966763 PMC4456647

[kiag349-B57] Yan J et al 2009. The Arabidopsis CORONATINE INSENSITIVE1 protein is a jasmonate receptor. Plant Cell. 21:2220–2236. 10.1105/tpc.109.065730. 19717617 PMC2751961

[kiag349-B58] Yang P, Lee Y, Szymanski DB, Xie J. 2025. Integrating CORUM and co-fractionation mass spectrometry to create enhanced benchmarks for protein complex predictions. Brief Bioinform. 26:bbaf154. 10.1093/bib/bbaf154. 40234106 PMC11998666

[kiag349-B59] Yu G et al 2020. A bacterial effector protein prevents MAPK-mediated phosphorylation of SGT1 to suppress plant immunity. PLoS Pathog. 16:e1008933. 10.1371/journal.ppat.1008933. 32976518 PMC7540872

[kiag349-B60] Yuan M, Ngou BPM, Ding P, Xin X-F. 2021. PTI-ETI crosstalk: an integrative view of plant immunity. Curr Opin Plant Biol. 62:102030. 10.1016/j.pbi.2021.102030. 33684883

[kiag349-B61] Zhou Y, Pan Q, Pires DEV, Rodrigues CHM, Ascher DB. 2023. DDMut: predicting effects of mutations on protein stability using deep learning. Nucleic Acids Res. 51:W122–W128. 10.1093/nar/gkad472. 37283042 PMC10320186

