# Supplementary material for: Discovery of regulatory complexes in Arabidopsis via protein–metabolite mapping during Pseudomonas infection
Source: Plant Physiol. 2026 Jun 9;201(2):kiag349. doi: 10.1093/plphys/kiag349 (PMC13317450; doi:10.1093/plphys/kiag349)

Supplementary Figure S1. NATA1 elution profile across control, infection and SAR PROMIS separation. The left panel (A) displays relative abundance, the right panel (B) displays normalized abundance.

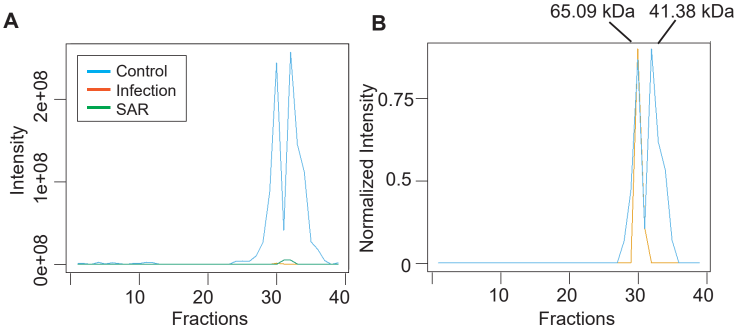

Supplementary Figure S2. NATA1 activity at 625 μM of putrescine. Bars show mean (n= 3) ± SEM. Statistical significance was assessed using an unpaired *t*-test, $P < 0.05$.

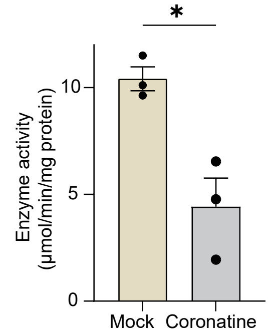

Supplementary Figure S3. Effects of the M45A substitution on NATA1 activity and dimerization. (A) Enzymatic activity assay of M45A. (B) MST-based dimerization assay of M45A. Error bars indicate SEM (n = 3).

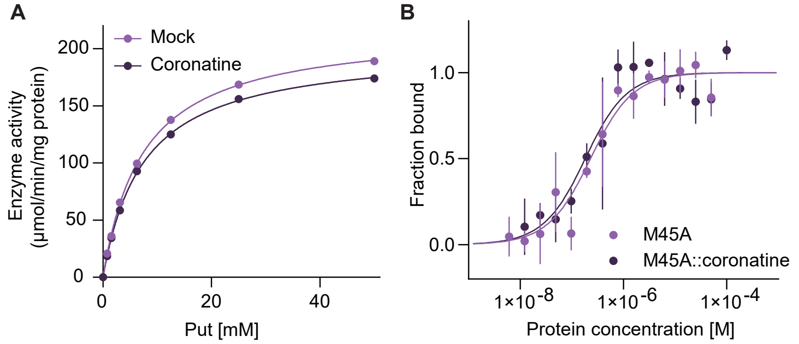

Supplementary Figure S4. Effects of the R214A and R183A substitutions on ligand binding and enzymatic activity. (A, C) MST assays of coronatine binding by WT, R214A, and R183A. (B, D) MST assays of acetyl-CoA binding by WT, R214A, and R183A. (E) Enzymatic activity of WT, R214A, and R183A. Error bars indicate SEM (n = 3).

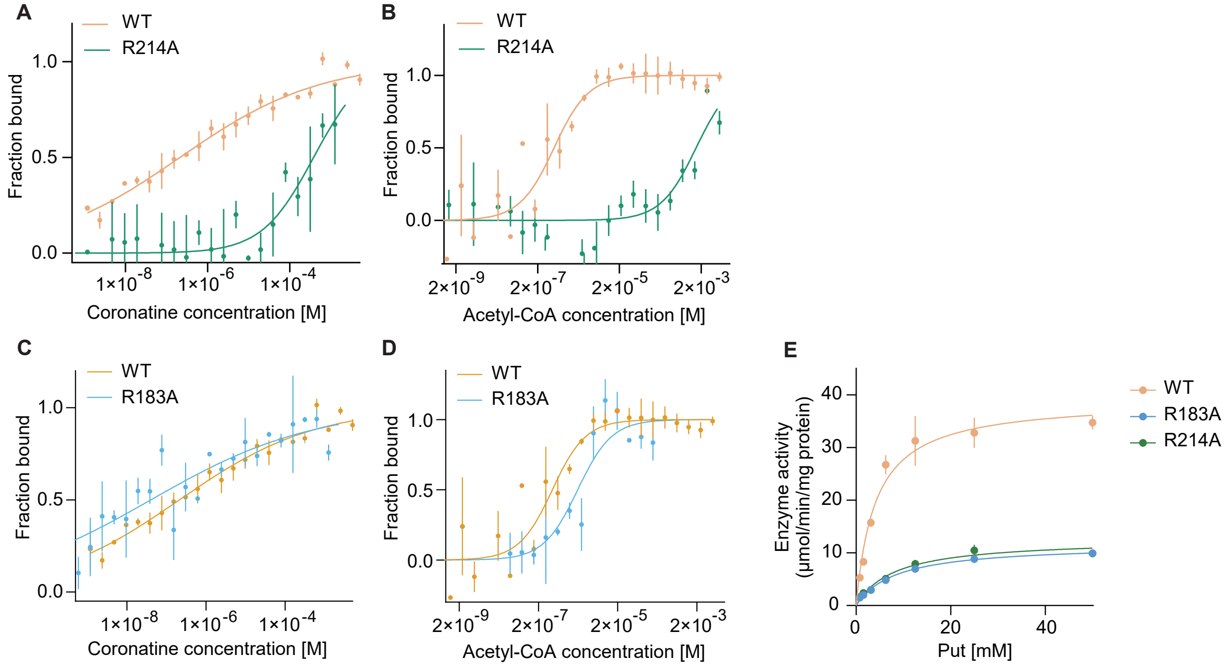

Supplementary Figure S5. Protein expression comparison of NATA1 split-luciferase fusion variants

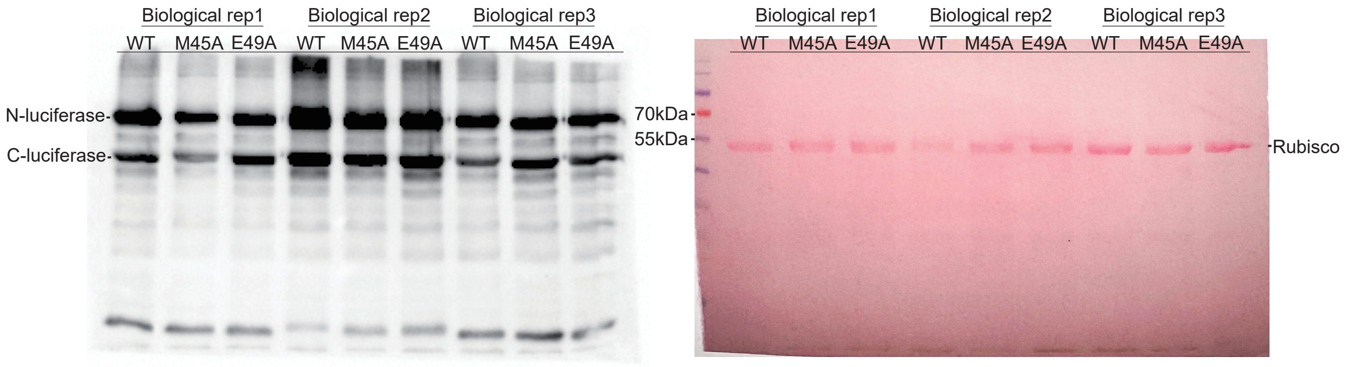

Supplement: kiag349_Supplementary_Data [file kiag349_supplementary_data.zip › Supplementary figure_revised.pdf]
