# Supplementary material for: Discovery of regulatory complexes in Arabidopsis via protein–metabolite mapping during Pseudomonas infection
Source: Plant Physiol. 2026 Jun 9;201(2):kiag349. doi: 10.1093/plphys/kiag349 (PMC13317450; doi:10.1093/plphys/kiag349)
Supplement: kiag349_Supplementary_Data [file kiag349_supplementary_data.zip › Supplementary Table.docx]

**Supplementary Table S1**. Primers used in this study.

| **Name** | **Seqeunce (5’-3’)** |
| --- | --- |
| M45A | AATCCACCAGGCGGCGGTTTTTG  AGCTTGTGAATGAAAGG |
| E49A | GGCGGTTTTTGCGCGCCTCACGC  ATCTGGTGGATTAGCTTGTG |
| R183A | GGGTGTCGGAgcgGTGGAGTGGATTG  AACTTCACTGCTTGCTTTG |
| R214A | GAGACTTTGTgcgCTCACTGGCG  CATTCCTTAAAAACCTGC |
| F_NATA1_GW | CACCATGGCGCCTCCAACCG |
| R_NATA1_no stop_GW | GATGTTTAGCTTGTCAATAGCTTGAAGT |
| R_NATA1_stop_GW | CTAGATGTTTAGCTTGTCAATAGCTTGA |
